# Spatial Modeling of COVID-19 Vaccine Hesitancy in the United States

**DOI:** 10.3390/ijerph18189488

**Published:** 2021-09-08

**Authors:** Abolfazl Mollalo, Moosa Tatar

**Affiliations:** 1 Department of Public Health and Prevention Science, School of Health Sciences, Baldwin Wallace University, Berea, OH 44017, USA; 2 Matheson Center for Health Care Studies, University of Utah, Salt Lake City, UT 84108, USA; moosa.tatar@utah.edu

**Keywords:** COVID-19, GIS, multiscale GWR, vaccination, vaccine hesitancy

## Abstract

Vaccine hesitancy refers to delay in acceptance or refusal of vaccines despite the availability of vaccine services. Despite the efforts of United States healthcare providers to vaccinate the bulk of its population, vaccine hesitancy is still a severe challenge that has led to the resurgence of COVID-19 cases to over 100,000 people during early August 2021. To our knowledge, there are limited nationwide studies that examined the spatial distribution of vaccination rates, mainly based on the social vulnerability index (SVI). In this study, we compiled a database of the percentage of fully vaccinated people at the county scale across the continental United States as of 29 July 2021, along with SVI data as potential significant covariates. We further employed multiscale geographically weighted regression to model spatial nonstationarity of vaccination rates. Our findings indicated that the model could explain over 79% of the variance of vaccination rate based on Per capita income and Minority (%) (with positive impacts), and Age 17 and younger (%), Mobile homes (%), and Uninsured people (%) (with negative effects). However, the impact of each covariate varied for different counties due to using separate optimal bandwidths. This timely study can serve as a geospatial reference to support public health decision-makers in forming region-specific policies in monitoring vaccination programs from a geographic perspective.

## 1. Introduction

The United States has reported over 35 million COVID-19 cases and more than 600 thousand COVID-19 deaths at the end of July 2021 [1], nearly one and a half years after the beginning of the global pandemic in March 2020 [2]. Various vaccines such as Pfizer-BioNTech, Moderna, and Johnson & Johnson are mass-produced and distributed in the United States. Previous studies indicated that the best way to control the pandemic is to vaccinate and immunize a large portion of the population to achieve herd immunity [3]. By the end of July 2021, almost 165 million people (~50.9% of the country’s total population) have been fully vaccinated against the virus, and 190 million people received at least one dose of one of those vaccines in this country. However, millions of people still have not been vaccinated, resulting in over 100 thousand new daily COVID-19 cases and 500 new daily COVID-19 deaths occurring during early August 2021 [4].

According to the World Health Organization (WHO), “vaccine hesitancy refers to delay in acceptance or refusal of vaccines despite the availability of vaccine services” [5]. The average acceptance rates of the COVID-19 vaccines are relatively low across the world, particularly in the Middle East, Russia, Africa, and several European countries. For example, Kuwait (23.6%), Jordan (28.4%), Italy (53.7%), and Russia (54.9%) have reported the lowest COVID-19 vaccine acceptance rates [6]. The United States also struggles with low COVID-19 acceptance rates (56.9%) [6], and approximately one-third of the South or Mountain West populations in this country are hesitant or unsure of getting the vaccine [7]. The primary reasons for vaccine hesitancy are low confidence in the efficacy of the COVID-19 vaccine, concerns regarding vaccine safety, the spread of misinformation, and mistrust of the government and public health system, especially among minorities [8,9]. Despite the efforts of the federal and state governments and numerous nationwide companies that have offered various COVID-19 vaccine incentives (e.g., lotteries, scholarships, and services) and rewards to increase vaccination rates [10], vaccine hesitancy is still one of the most crucial barriers to control the COVID-19 pandemic.

Prior research has indicated a significant impact of socioeconomic determinants on becoming immunized against Influenza regardless of pre-existing risk factors [11]. A recent study also found that being younger and income loss during the pandemic were significantly associated with refusal and delay in immunization against the COVID-19 virus [9]. Another study explored the spatial relationship between COVID-19 incidence and environmental, socioeconomic, and demographic variables and found that income inequality could explain a considerable variance of COVID-19 incidence in the United States [12]. However, research on the COVID-19 vaccine hesitancy in the United States is not exhaustive, and inadequate studies have examined the role of specific social vulnerabilities for COVID-19 vaccination rates [13]. Social Vulnerability Index (SVI), developed by the Centers for Disease Control and Prevention (CDC), assesses the resilience of communities against infectious disease outbreaks who are at risk of being impacted by any public health crisis [14]. The SVI consists of socioeconomic status, household composition, minority status, housing type, and transportation [15]. This study used SVI data and geospatial modeling techniques to investigate the spatial pattern of COVID-19 vaccination hesitancy in the United States. Several geospatial models have previously been used to analyze and model COVID-19 cases [16], infection [17], case fatality ratio [18], and mortality [19].

Recent vaccination statistics imply a higher social and racial disparity in regions with fewer vaccination rates [20]. To our knowledge, this is the first spatial epidemiological study that investigates the socioeconomic determinants of the COVID-19 vaccination rate in the continental United States. The findings of this nationwide study can enhance county-level policies in controlling the ongoing spread of disease and reinforce public health decisions from geospatial perspective. This timely study seeks to understand spatial patterns of COVID-19 vaccinations and identify potential socioeconomic determinants that might prevent or promote the COVID-19 vaccination rate in the United States.

## 2. Materials and Methods

### 2.1. Study Settings

This geographic system information (GIS)-based retrospective study included a database of COVID-19 vaccination rates as the response variable and SVI data as potential significant covariates across the continental United States. We investigated the county-level (*n* = 3103) impacts of socioeconomic determinants on the COVID-19 vaccination rate for fully vaccinated people. The COVID-19 vaccination rates were collected up to 29 July 2021 from the Bansal Lab at Georgetown University [21]. The Bansal Lab website has integrated vaccination rates from the CDC Tracker Data website [22] and individual state health departments at the county level across the United States.

We also downloaded the latest available SVI data from the CDC website [23], released in 2018. We used 15 social vulnerability variables as potential covariates in four main themes:

(1) socioeconomic status: percentage of people below poverty, unemployment rate, per capita income, percentage of people with no high school diploma,
(2) household composition and disability: percentage of people aged 65 and older, percentage of people aged 17 and younger, percentage of non-institutionalized people with a disability, percentage of single-parent households with children,
(3) minority status and language: percentage of minority people (except white and non-Hispanic),
(4) housing type and transportation: percentage of housing in structures with 10+ units, percentage of mobile homes, percentage of over-occupied housing units, percentage of households with no vehicle available, and percentage of institutionalized group quarters (e.g., correctional institutions, nursing homes).

We computed population density per square mile for each county and also included the percentage of uninsured people in the total civilian non-institutionalized population as other covariates (Table 1). The shapefiles of state and county boundaries were obtained from the US Census TIGER/Line website [24] for further geospatial analysis. All data are freely available from the above resources.

The following sections will identify potential significant covariates and examine their impacts on the COVID-19 vaccination rate, based on three different models. The employed models include a global ordinary least squares (OLS) as a baseline model and two local models: geographically weighted regression (GWR) and multiscale GWR (MGWR). We calibrated the models and evaluated the models’ accuracy in explaining the variance of COVID-19 vaccination rate in the continental United States.

### 2.2. Ordinary Least Squares Model (OLS)

The traditional ordinary least squares (OLS) regression model was fitted as the baseline model. This global model is defined as [25]:

(1) $$y_{i}=\beta_{0}+\beta x_{i}+\varepsilon_{i}$$

where $y_{i}$ is the percentage of fully vaccinated people against COVID-19 at county i; $\beta_{0}$ is the intercept; β is the vector of the estimated coefficients of covariates; $x_{i}$ is the vector of selected covariates, and $\varepsilon_{i}$ is the error term in the model estimates.

To calibrate the OLS model, we scrutinized and removed redundant covariates. First, among all potential covariates, we only selected covariates with the lowest Pearson product correlation coefficient with others (|r| < 0.4). Moreover, the OLS model was selected based on stepwise forward regression that captures the maximum variance (increased R^2^ the most) and indicated little multicollinearity for each covariate (variance inflation factor (VIF) < 3). Finally, the insignificant covariates were excluded from the OLS model (*p* < 0.05).

However, it should be noted that the OLS model suffers from two main assumptions. First, OLS assumes spatial stationarity or homogeneity across the study area, while the relationship between the response variable and the covariates can vary in different counties (spatial heterogeneity). Moreover, OLS assumes that the model’s residuals should not be spatially autocorrelated [26].

### 2.3. Geographically Weighted Regression (GWR)

To relax OLS assumptions, the geographically weighted regression (GWR) model was employed. Unlike OLS, GWR allows coefficients of each covariate to vary for different counties across the United States, and thus GWR can capture spatial heterogeneity [27]. This local model uses an adaptive kernel (the same number of observations) to borrow a subset of data from nearby counties to estimate model parameters at any county in the United States [28].

The GWR model can be formulated as:

(2) $$y_{i}=\beta_{i0}+\overset{M}{\underset{j=1}{\sum }}\beta_{ij}X_{ij}+\varepsilon_{i}i=1,\;2,\;..,\;M$$

where $y_{i}$ is the percentage of fully vaccinated people against COVID-19 at county i; $\beta_{i0}$ is the intercept for county i; $\beta_{ij}$ is the estimation of coefficient for jth covariate; $X_{ij}$ is the jth covariate at county i; M is the number of covariates, and $\varepsilon_{i}$ is the error term in the model estimates.

Although GWR addresses spatial heterogeneity, there are several limitations in using this model [29]. First, the model applies a single and uniform spatial scale (bandwidth) for all covariates. In other words, GWR disregards the possibility that the covariates affecting the COVID-19 vaccination rate are often at different spatial scales, which might bias the result by exaggerating or underestimating the contribution of each covariate. Moreover, the presence of local multicollinearity can cause instability of the parameter estimates [30]. To date, GWR has been applied to several COVID-19 morbidity and mortality studies [31,32,33,34,35]. However, to our knowledge, GWR has never been used in the geospatial modeling of COVID-19 vaccination rates.

### 2.4. Multiscale Geographically Weighted Regression (MGWR)

To overcome GWR drawbacks, Fotheringham et al. (2017) developed an extension of GWR: multiscale GWR (MGWR) [29]. MGWR allows the relationship between covariates and the COVID-19 vaccination to vary locally using separate bandwidths for each covariate. Moreover, MGWR can alleviate the multicollinearity problem by minimizing the overfitting and concurvity of GWR, leading to more reliable model estimates [30]. Therefore, we refined GWR by applying MGWR to examine the effects of scale for each covariate on the COVID-19 vaccination rate. Using the same notation as in Equation (2), the model can be expressed as follows:

(3) $$y_{i}=\overset{M}{\underset{j=1}{\sum }}\beta_{bwj}X_{ij}+\varepsilon_{i}\;$$

where $\beta_{bwj}$ is the estimation of coefficient for county i in which bwj is the jth optimal bandwidth [29]. MGWR uses an iterative back-fitting algorithm using standard GWR estimates and tests the goodness of fit for each covariate. Bandwidths control weighting intensity or data-borrowing used by both GWR and MGWR. These models were calibrated based on adaptive bandwidth using a bi-square kernel for weighting the data included within the bandwidth [36]. Similar to GWR, in MGWR, optimal bandwidths were determined using the corrected Akaike Information Criterion (AICc). The MGWR model has been used in the study of COVID-19 at the national and global levels [16,18,36].

### 2.5. Model Evaluation

To obtain scale-free bandwidths, all covariates and the response variable were converted to standardized z-score (mean = 0, standard deviation = 1). This standardization operation can also facilitate direct comparison of bandwidths and reduce computational runtime for local models [29]. MGWR 2.2 software that is available from [37] was used to run all models based on the same significant covariates obtained from the OLS model.

We further compared the performance of models (i.e., OLS, GWR, and MGWR) based on the combination of criteria: Adj. R^2^, AICc, residual sum of square (RSS), and multicollinearity. A larger adjusted R^2^ indicates the model can explain a larger variance of COVID-19 vaccination rate and is preferred. While the lower values for AICc and RSS are desired, these values imply the most parsimonious model and the amount of variance that the model could not explain, respectively [38].

Global Moran’s *I* statistic was employed on the model’s residuals to determine whether the residuals are spatially autocorrelated [39,40]. A significant spatial autocorrelation among residuals indicates the model is missing key covariates [41,42]. To test local multicollinearity, we computed the local condition numbers for GWR and MGWR. The local condition number between 15 and 30 or above are problematic and imply multicollinearity [38]. We further mapped local condition numbers for GWR and MGWR to visually check local multicollinearity. Moreover, local R^2^ values were mapped to compare the goodness of fit spatial distributions for GWR and MGWR models.

After identifying the optimal model, we joined the estimations obtained from the model to the corresponding county shapefile and mapped the coefficients to depict the local effects of each covariate on the COVID-19 vaccination rate in the United States. All maps were generated in ArcGIS Desktop 10.7 (ESRI, Redlands, CA).

## 3. Results

**Figure 1:**
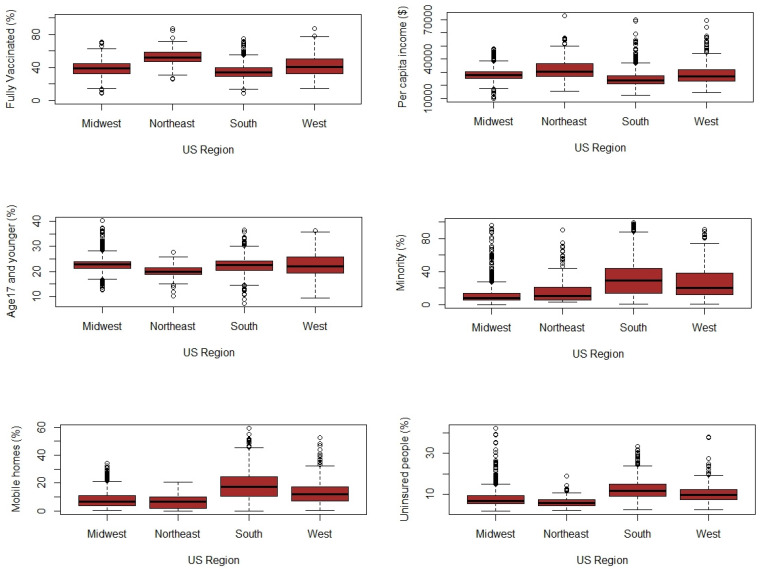
Boxplots of the response variable and covariates used in the models.

Preliminary results indicated that as of 29 July 2021, on average, 47.25% of people were fully vaccinated against the virus across the continental United States (range: 8.9–87.4%). Among all counties, 428 counties (13.8% of total counties) had reported over 50% (full) vaccination rates. In comparison, four counties (in Arkansas, North Dakota, South Dakota, and Nebraska states) had less than 10% vaccination rates, while three counties in Massachusetts (2 counties) and Colorado had reported above 80% (full) vaccination rates. The full vaccination rates were higher in the Northeast region of the United States, with generally better socioeconomic status. We classified US counties based on four regions: West, Midwest, South, and Northeast. We then used a one-way analysis of variance (ANOVA) test to examine if there were significant differences in the means of vaccination rates. Results of the ANOVA test indicate that only the Northeast region had a significantly higher mean full vaccination rate compared to the other three regions (*p* < 0.05) (Figure 1). Figure 1 demonstrates the boxplot for the used covariates for US regions. Table 2 summarizes the mean values of the COVID-19 vaccination rates and covariates used in the models classified based on US regions.

The final OLS model was constructed based on five statistically significant covariates. According to Table 3, Per capita income and Minority (%) had positive relationships with the COVID-19 vaccination rate, while Age 17 and younger (%), Mobile homes (%), and Uninsured people (%) indicated negative associations with the response variable (*p* < 0.01). The VIF values for all covariates were <2, indicating that multicollinearity is not a severe problem. However, the residuals were highly clustered (Moran’s *I* = 0.30, z-score = 58.09, *p*-value < 0.01), suggesting that the model overfitted in some counties and under fitted in some other counties. The autocorrelated residuals in OLS violate the independence of errors assumption; thus, the coefficient estimates should be interpreted with caution.

Compared to OLS, both local models yielded better fits with improving adjusted R^2^ by at least 32%, which means that the local models could explain at least 77.7% of the variance of COVID-19 vaccination rates in the United States. However, MGWR showed a slightly better fit (Adj. R^2^ = 79.1) than GWR (Adj. R^2^ = 77.7). The MGWR was also the most parsimonious model (AICc = 4437.25) compared to GWR (AICc = 4676.53), while the OLS was the least parsimonious model (AICc = 6954.02). Similarly, MGWR produced the lowest residual sum of squares (RSS = 569.38), followed by GWR (RSS = 598.47) and OLS (RSS = 1697.984). The Moran’s *I* indicated that the residuals of GWR had a clustered distribution but with less intensity than OLS (GWR Moran’s *I* = 0.01, z-score = 2.77, *p* < 0.05). In contrast, the distribution of residuals in MGWR was dispersed (Moran’s *I* = –0.02, z-score = –3.5, *p* < 0.05). Table 4 summarizes the evaluation metrics for all models.

**Figure 2:**
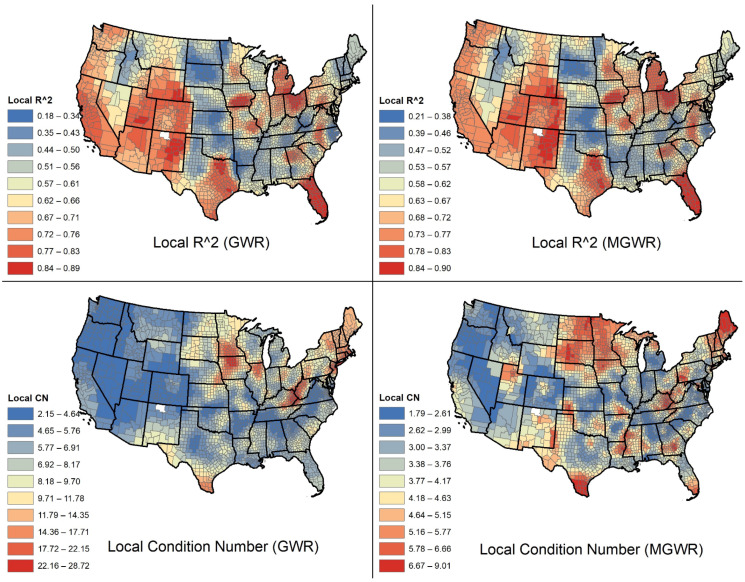
Spatial distribution of local R^2^ and local condition numbers for GWR (left column) and MGWR (right column) models.

The local R^2^ of MGWR ranged between 20.8% and 90.5% (median = 63.5%, standard deviation = 13.26%) compared with the local R^2^ of the GWR model ranging between 18.3% and 89.4% (median = 60.8%, standard deviation = 13.8%). This indicates that MGWR achieved a better local fit than GWR. Figure 2 shows the geographic distribution of local R^2^ for both models. According to this map, the models have the best fit in Texas, Florida, Michigan, Ohio, and several counties in the Western United States. Moreover, the local condition numbers in MGWR were between 1.72 and 8.63 (median = 3.52, standard deviation = 1.05), while these numbers ranged between 2.15 and 28.7 for GWR. Since the usual value for the local condition number is between 15 and 30, there is a possible indication of multicollinearity in GWR. Figure 2 depicts the spatial distribution of local condition numbers for both local models.

**Figure 3:**
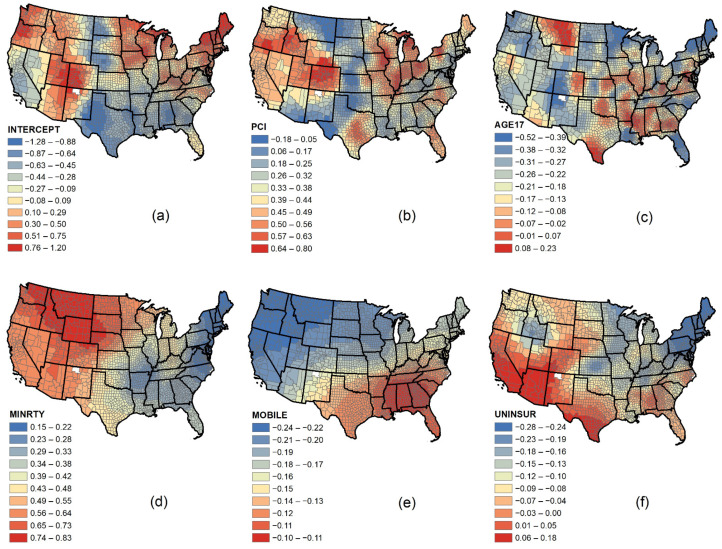
The effects of (**a**) Intercept; (**b**) Per capita income; (**c**) Age 17 and younger (%); (**d**) Minority (%); (**e**) Mobile homes (%), and (**f**) Uninsured people (%) on COVID-19 vaccination rate (as of 29 July 2021) in the United States, obtained from MGWR model.

The GWR model yielded an optimal bandwidth of 104, showing the number of counties with non-zero weighting in the model calibration. In comparison, MGWR produced distinct bandwidths for each covariate. Table 5 provides optimal bandwidths pertaining to each covariate, effective number of parameters, and the adjusted critical t-values for significance testing at a 95% confidence interval. Bandwidth confidence intervals were also measured at a 95% confidence level. Almost all t-values are higher (more conservative) than conventional t-value. The t-values for Age 17 and younger (%) from MGWR are larger than the corrected t-value from GWR of 3.39 (i.e., more conservative), while others are smaller (i.e., less conservative). The large bandwidth for Mobile homes (%) indicates that this covariate has the largest scale of influence than the others, while Per capita income and Age 17 and younger had the most local impacts as indicated by their smaller bandwidths (Figure 3).

According to Figure 3, Minority (%) positively impacted the vaccination rate that steadily increased from East to West. Conversely, Uninsured people (%) had a negative impact on the vaccination rate that also increased from East to West. Mobile homes (%) showed a constant negative impact that steadily decreased from North to South. Both Per capita income and Age 17 and younger (%) indicated more local effects than the other covariates. Per capita income had the highest impacts in Midwest and Western regions. In contrast, Age 17 and younger (%) had a steadily positive association with vaccination rates, with the most effects mainly in South-central areas and Montana.

## 4. Discussion

WHO has classified vaccine hesitancy as one of the top-ranked crucial global health threats in 2019 due to the resurgence of vaccine-preventable diseases and the declining vaccination rates [43]. This GIS-based retrospective study aimed to analyze spatial heterogeneity of COVID-19 full vaccination rates across all counties in the continental United States using SVI data. We used 15 SVI covariates in four themes, including socioeconomic status, household composition and disability, minority status and language, and housing type and transportation, in addition to population density and percentage of uninsured people at the county scale. The OLS model could not account for spatial variations of vaccination rates, as its residuals were highly autocorrelated, emphasizing the necessity of using local models. With fewer assumptions about data, the local models could explain nearly 80% (MGWR Adj. R^2^ = 79.1 and GWR Adj. R^2^ = 77.7) of the variances of COVID-19 vaccination rates. The results of the MGWR model, as the best-fitted model in this study, showed that the model fitted well in most areas of New Mexico, Colorado, Utah, Wyoming, Florida, Ohio, Michigan, and Northeast Texas based on Per capita income, Age 17 and younger (%), Minority (%) (except white and non-Hispanic), Mobile homes (%), and Uninsured people (%). However, the model was mainly under-fitted in most counties of South Dakota, Kansas, Oklahoma, and Mississippi, which also had reported lower vaccination rates. The poor performance of the model in counties with low vaccination rates can indicate evidence for missing key covariates such as environmental, demographic, and health-related variables. However, these covariates were outside of the research objectives.

Our findings confirmed the strengths of MGWR compared to the GWR model not only because of a more accurate model fit with fewer covariates but also due to addressing the scale issue. In other words, instead of using a single average bandwidth for all covariates in the widely used GWR model, which could mask the actual spatial variations, MGWR provided separate optimal scales for each covariate. Although distinct bandwidth significantly increased the computational complexity of the model, it better revealed the spatial pattern of the effect of each covariate on the COVID-19 vaccination rate. For instance, the larger bandwidth (e.g., Mobile home (%)) indicated a more stable relationship, and smaller bandwidth (e.g., Age 17 and younger) suggested a more local association with COVID-19 vaccination rates.

Vaccine hesitancy among ethnic minorities might be rooted in the history of racial injustices and their negative experiences with the culturally insensitive healthcare system, which has adversely impacted the COVID-19 vaccine uptake in the United States [44]. Some studies have reported a significant portion of vaccine hesitancy, particularly among blacks, women, and conservatives in the United States [45,46]. However, based on the SVI definition of minorities (all persons except white, non-Hispanic), we found a positive impact of minorities on COVID-19 vaccination rates, particularly a higher positive rate in the West and the Northwestern United States than in Eastern counties. The inconsistency between our findings and [45,46] regarding the vaccination rates among minorities might be due to the differences in the definition of minorities and the scale of analysis. Thus, further study should investigate the heterogeneity between minority groups. Prioritizing these underserved and vulnerable communities as well as allocating health resources based on their population size may avoid increasing existing health disparity in the United States.

Our results indicated no relationship between the percentage of persons with no high school diploma and vaccination rates at the county level. This finding was consistent with an early survey in 2016 and 2017 on vaccine hesitancy in low- and middle-income countries, including China, India, Bangladesh, Ethiopia, and Guatemala [47]. However, there is no consensus about the relationship. A recent scoping review [48] in high-income countries found that lower education level is positively associated with COVID-19 vaccine hesitancy. A hypothesis that may explain this association is people without a college degree may underestimate effectiveness or overestimate the risks of vaccines, compared to the educated population who are more likely to distinguish authentic information from misinformation.

Our findings indicated that Per capita income and the Uninsured rate were positively and negatively associated with vaccination rates, respectively. However, the strengths of associations significantly varied by geographic location. This agrees with the published data of CDC that indicates in states with higher median household incomes, a higher vaccination rate is reported. For instance, in Maryland and New Jersey (with higher median incomes), over 70% of populations have been vaccinated, while at the same time, in states such as Mississippi and Arkansas with lower household income, only <30% of the populations have been fully vaccinated. A recent US Census Bureau Household plus survey [49] shows that most unvaccinated Americans live in households that make less than $50,000 annually. Moreover, a study by Lindemer et al. [50] suggests that the US counties with lower insurance coverage have significantly slower vaccine rollout. A possible explanation may be the fear of the uninsured population of receiving a bill, even though the vaccines are free to the public in the United States.

Living in non-deprived neighborhoods can generally provide better health status due to higher access to health care and social resources [51]. However, nearly 20 million residents in the United States live in mobile homes, and over half of the mobile homes are in rural areas [52]. This population is more likely to have environmentally related diseases and can face health disparities [53]. Having a lower COVID-19 vaccination rate and less immunity against the COVID-19 virus can exacerbate the adverse health outcomes associated with their neighborhood environment (e.g., access to care, insurance coverage, and financial situation). Moreover, our findings implied that the counties with a higher proportion of uninsured individuals had lower COVID-19 vaccination rates. Although effective preventive services may reduce health concerns, uninsured adults receive significantly lower preventive services than insured people [54].

There are several caveats and inherent limitations in this study. First, due to the highly dynamic nature of the disease, such as the highly contagious delta variant and continuous change of the vaccination statistics, follow-up studies with more recent data are required to provide up-to-date information for policymakers to fight against the disease. Moreover, the findings of this study should only be explained at the county scale. In other words, sub-county and individual inferences can be misleading due to ecological fallacy. In addition, model results can change for different spatial units due to modifiable areal unit problems. Thus, for future studies, conducting higher-resolution spatial analysis at multiple scales together with incorporating environmental, demographic, and health-related variables is recommended. Moreover, local healthcare facilities and available social resources that can potentially impact the vaccination rate in different counties require further investigations. Finally, age-specific modeling could not be implemented due to the lack of data for many states.

## 5. Conclusions

In summary, our findings reveal that the geospatial disparity of COVID-19 vaccine hesitancy in the United States is highly associated with socio-economics covariates. Therefore, targeted interventions on potentially modifiable socioeconomic social vulnerability factors faced by race/ethnic minorities, especially in underserved and vulnerable communities, may maximize vaccine uptake. Policymakers should also increase public trust and participation by engaging community groups, champions, and faith leaders to reduce vaccine hesitancy rates [8]. Moreover, wider communication through media and fighting against widespread misinformation would raise public awareness about the scale and consequences of the COVID-19 pandemic. Evidence-based and scientific benefits of the COVID-19 vaccine may also change the perception of the COVID-19 vaccination at both individual and organizational levels as well as maximize COVID-19 vaccine acceptance. To the authors’ knowledge, there is a lack of research on spatial modeling of COVID-19 vaccination in the United States; thus, this timely study can serve as a geospatial reference to support public health decision-makers in forming region-specific policies in monitoring vaccination programs.

## Figures and Tables

**Table 1:** The covariates used in this study [14].

No.	Covariate	Abbreviation	Definition
1	Below poverty %	POV	Percentage of persons below federal poverty level
2	Unemployment rate %	UNEMP	Number of persons who are unemployed but seeking a job
3	Per capita income	PCI	Per capita annual income in dollars
4	No high school diploma %	NOHSDP	Percentage of persons with no high school diploma (age 25+)
5	Age 65 and older %	AGE65	Percentage of persons aged 65 and older
6	Age 17 and younger %	AGE17	Percentage of persons aged 17 and younger
7	Non-institutionalized with a disability %	DISABL	Percentage of civilian non-institutionalized population with a disability
8	Single-parent households with children %	SNGPNT	Percentage of single-parent households with children under 18
9	Minority (except white, non-Hispanic) %	MINRTY	Percentage minority (all persons except white, non-Hispanic)
10	Age 5+ who speak limited English %	LIMENG	Percentage of persons (age 5+) who speak English “less than well” estimate
11	Housing in structures with 10+ units %	MUNIT	Percentage of housing structures with 10 or more units out of all residential housing types
12	Mobile homes %	MOBILE	Percentage of mobile homes out of all residential housing types
13	Over-occupied housing units %	CROWD	Percentage of occupied housing units with more occupants than number of rooms
14	Households with no vehicle available %	NOVEH	Percentage of households with no vehicle ownership
15	Institutionalized group quarters %	GROUPQ	Percentage of persons residing in institutionalized group quarters (e.g., correctional institutions, nursing homes)
16	Uninsured people %	UNISUR	Percentage uninsured in the total civilian non-institutionalized population
17	Population density per square mile	POPDEN	Number of persons per square mile

**Table 2:** Mean values of the response variable and covariates used in the models classified based on regions in the United States as of 29 July 2021.

US Region	Fully Vaccinated (%)	Per Capita Income ($)	Age 17 and Younger (%)	Minority (%)	Mobile Homes (%)	Uninsured People (%)
West	51.22	28,274	22.63	27.29	13.12	10.08
Midwest	45.51	28,127	22.66	11.84	7.96	7.84
South	42.41	24,875	22.39	31.31	17.76	12.18
Northeast	54.86	32,605	19.97	16.60	6.36	6.09

**Table 3:** Results of OLS model of COVID-19 vaccination rates in the United States.

Covariate	Coefficient (EST.)	SE	T (EST/SE)	*p*-Value	VIF
Intercept	0.000	0.013	0.000	1.000	–
Per capita income	0.360	0.017	21.446	0.000	1.599
Age 17 and younger (%)	–0.244	0.015	–16.643	0.000	1.217
Minority (%)	0.338	0.016	21.471	0.000	1.408
Mobile homes (%)	–0.259	0.017	–15.510	0.000	1.587
Uninsured people (%)	–0.190	0.018	–10.761	0.000	1.763

**Table 4:** Comparison of OLS, GWR, and MGWR models of COVID-19 vaccination rates in the United States.

	Model		
Evaluation Statistic	OLS	GWR	MGWR
AICc	6954.21	4676.526	4437.25
Adj. R^2^	45.3	77.7	79.1
RSS	1697.984	598.47	569.38
Log-Likelihood	−3469.992	–1849.97	–1772.57

**Table 5:** Comparison of bandwidths, the effective number of parameters, and critical t-values for GWR and MGWR models.

	Bandwidth (95% CI)		Effective Number of Parameters		Critical t-Value (95%)	
	GWR	MGWR	GWR	MGWR	GWR	MGWR
Model	n/a	n/a	420.840	388.934	3.388	n/a
Intercept	104 (98, 107)	44 (44, 46)	*n/a*	181.954	*n*/a	3.642
Per capita income	104 (98, 107)	95 (88, 107)	*n/a*	69.082	*n*/a	3.384
Age 17 and younger (%)	104 (98, 107)	74 (67, 82)	*n/a*	98.274	*n*/a	3.48
Minority (%)	104 (98, 107)	322 (278, 384)	*n/a*	14.508	*n*/a	2.927
Mobile homes (%)	104 (98, 107)	1283 (1042, 1936)	*n/a*	3.77	*n*/a	2.478
Uninsured people (%)	104 (98, 107)	245 (213, 278)	*n/a*	21.345	*n*/a	3.046

## Data Availability

The data presented in this study are openly available in [21,23,24] references.

## References

[1] Center for Systems Science and Engineering (CSSE) (2021). Global Cases by the Center for Systems Science and Engineering (CSSE) at Johns Hopkins University (JHU). https://github.com/CSSEGISandData/COVID-19.

[2] World Health Organization (2020). Coronavirus Disease 2019 (COVID-19) Situation Report-51. https://www.who.int/docs/default-source/coronaviruse/situation-reports/20200311-sitrep-51-covid-19.pdf?sfvrsn=1ba62e57_10.

[3] Lurie N., Sharfstein J.M., Goodman J.L. (2020). The development of COVID-19 vaccines: Safeguards needed. JAMA.

[4] Centers for Disease Control and Prevention (2021). Trends in Number of COVID-19 Cases and Deaths in the US Reported to CDC, by State/Territory. COVID Data Tracker. https://covid.cdc.gov/covid-data-tracker/#trends_dailytrendscases.

[5] MacDonald N.E. (2015). Vaccine hesitancy: Definition, scope and determinants. Vaccine.

[6] Sallam M. (2021). COVID-19 vaccine hesitancy worldwide: A concise systematic review of vaccine acceptance rates. Vaccines.

[7] Centers for Disease Control and Prevention (2021). Estimates of Vaccine Hesitancy for COVID-19. https://data.cdc.gov/stories/s/Vaccine-Hesitancy-for-COVID-19/cnd2-a6zw.

[8] Razai M.S., Osama T., McKechnie D.G., Majeed A. (2021). Covid-19 Vaccine Hesitancy among Ethnic Minority Groups. BMJ.

[9] Soares P., Rocha J.V., Moniz M., Gama A., Laires P.A., Pedro A.R., Nunes C. (2021). Factors associated with COVID-19 vaccine hesitancy. Vaccines.

[10] National Governors Association (2021). COVID-19 Vaccine Incentives. Publications. https://www.nga.org/center/publications/covid-19-vaccine-incentives/.

[11] Endrich M.M., Blank P.R., Szucs T.D. (2009). Influenza vaccination uptake and socioeconomic determinants in 11 European countries. Vaccine.

[12] Mollalo A., Vahedi B., Rivera K.M. (2020). GIS-based spatial modeling of COVID-19 incidence rate in the continental United States. Sci. Total Environ..

[13] Mollalo A., Rivera K.M., Vahedi B. (2020). Artificial neural network modeling of novel coronavirus (COVID-19) incidence rates across the continental United States. Int. J. Environ. Res. Public Health.

[14] Flanagan B.E., Gregory E.W., Hallisey E.J., Heitgerd J.L., Lewis B. (2011). A social vulnerability index for disaster management. J. Homel. Secur. Emerg. Manag..

[15] Centers for Disease Control and Prevention (2018). CDC’s Social Vulnerability Index (SVI). https://svi.cdc.gov/.

[16] Iyanda A.E., Adeleke R., Lu Y., Osayomi T., Adaralegbe A., Lasode M., Osundina A.M. (2020). A retrospective cross-national examination of COVID-19 outbreak in 175 countries: A multiscale geographically weighted regression analysis (January 11-June 28, 2020). J. Infect. Public Health.

[17] Iyanda A.E., Boakye K.A., Lu Y., Oppong J.R. (2021). Racial/Ethnic Heterogeneity and Rural-Urban Disparity of COVID-19 Case Fatality Ratio in the USA: A Negative Binomial and GIS-Based Analysis. J. Racial Ethn. Health Disparities.

[18] Al Kindi K.M., Al-Mawali A., Akharusi A., Alshukaili D., Alnasiri N., Al-Awadhi T., El Kenawy A.M. (2021). Demographic and socioeconomic determinants of COVID-19 across Oman-A geospatial modelling approach. Geospat. Health.

[19] Vahabi N., Salehi M., Duarte J.D., Mollalo A., Michailidis G. (2021). County-level longitudinal clustering of COVID-19 mortality to incidence ratio in the United States. Sci. Rep..

[20] Iyanda A., Boakye K., Lu Y. (2021). COVID-19: Evidenced Health Disparity. Encyclopedia.

[21] Bansal Lab. http://www.vaccinetracking.us/.

[22] COVID CDC Tracker. https://covid.cdc.gov/covid-data-tracker/#vaccinations-county-view.

[23] CDC/ATSDR SVI Data. https://www.atsdr.cdc.gov/placeandhealth/svi/data_documentation_download.html.

[24] TIGER/Line. https://www.census.gov/geographies/mapping-files/time-series/geo/tiger-line-file.html.

[25] Hutcheson G.D., Moutinho L., Hutcheson G.D. (2011). Ordinary least-squares regression. The SAGE Dictionary of Quantitative Management Research.

[26] Anselin L., Arribas-Bel D. (2013). Spatial fixed effects and spatial dependence in a single cross-section. Pap. Reg. Sci..

[27] Tu J., Xia Z.G. (2008). Examining spatially varying relationships between land use and water quality using geographically weighted regression I: Model design and evaluation. Sci. Total Environ..

[28] Brunsdon C., Fotheringham A.S., Charlton M.E. (1996). Geographically weighted regression: A method for exploring spatial nonstationarity. Geogr. Anal..

[29] Fotheringham A.S., Yang W., Kang W. (2017). Multiscale geographically weighted regression (MGWR). Ann. Am. Assoc. Geogr..

[30] Oshan T.M., Smith J.P., Fotheringham A.S. (2020). Targeting the spatial context of obesity determinants via multiscale geographically weighted regression. Int. J. Health Geogr..

[31] Chen Y., Jiao J. (2020). Relationship between Socio-Demographics and COVID-19: A Case Study in Three Texas Regions. https://papers.ssrn.com/sol3/papers.cfm?abstract_id=3636484.

[32] Wu X., Zhang J. (2021). Exploration of spatial-temporal varying impacts on COVID-19 cumulative case in Texas using geographically weighted regression (GWR). Environ. Sci. Pollut. Res..

[33] Maiti A., Zhang Q., Sannigrahi S., Pramanik S., Chakraborti S., Cerda A., Pilla F. (2021). Exploring spatiotemporal effects of the driving factors on COVID-19 incidences in the contiguous United States. Sustain. Cities Soc..

[34] Horse AJ Y., Yang T.C., Huyser K.R. (2021). Structural inequalities established the architecture for COVID-19 pandemic among native Americans in Arizona: A geographically weighted regression perspective. J. Racial Ethn. Health Disparities.

[35] Zhang J., Wu X., Chow T.E. (2021). Space-Time Cluster’s Detection and Geographical Weighted Regression Analysis of COVID-19 Mortality on Texas Counties. Int. J. Environ. Res. Public Health.

[36] Mansour S., Al Kindi A., Al-Said A., Al-Said A., Atkinson P. (2021). Sociodemographic determinants of COVID-19 incidence rates in Oman: Geospatial modelling using multiscale geographically weighted regression (MGWR). Sustain. Cities Soc..

[37] Oshan T.M., Li Z., Kang W., Wolf L.J., Fotheringham A.S. (2019). Mgwr: A Python Implementation of Multiscale Geographically Weighted Regression for Investigating Process Spatial Heterogeneity and Scale. ISPRS Int. J. Geo-Inf..

[38] Arbona S.I., Barro A.S. (2020). Peer Reviewed: Exploring the Spatial Determinants of Late HIV Diagnosis in Texas. Prev. Chronic Dis..

[39] Mollalo A., Alimohammadi A., Shirzadi M.R., Malek M.R. (2015). Geographic information system-based analysis of the spatial and spatio-temporal distribution of zoonotic cutaneous leishmaniasis in Golestan Province, north-east of Iran. Zoonoses Public Health.

[40] Mollalo A., Rivera K.M., Vahabi N. (2021). Spatial statistical analysis of pre-existing mortalities of 20 diseases with COVID-19 mortalities in the continental United States. Sustain. Cities Soc..

[41] Iyanda A.E., Osayomi T. (2020). Is there a relationship between economic indicators and road fatalities in Texas? A multiscale geographically weighted regression analysis. GeoJournal.

[42] Mollalo A., Vahedi B., Bhattarai S., Hopkins L.C., Banik S., Vahedi B. (2020). Predicting the hotspots of age-adjusted mortality rates of lower respiratory infection across the continental United States: Integration of GIS, spatial statistics and machine learning algorithms. Int. J. Med Inform..

[43] World Health Organization (2019). Ten Threats to Global Health in 2019. https://www.who.int/news-room/spotlight/ten-threats-to-global-health-in-2019.

[44] Hildreth J.E., Alcendor D.J. (2021). Targeting COVID-19 Vaccine Hesitancy in Minority Populations in the US: Implications for Herd Immunity. Vaccines.

[45] Callaghan T., Moghtaderi A., Lueck J.A., Hotez P.J., Strych U., Dor A., Motta M. (2020). Correlates and Disparities of COVID-19 Vaccine Hesitancy. https://papers.ssrn.com/sol3/papers.cfm?abstract_id=3667971.

[46] Nguyen L.H., Joshi A.D., Drew D.A., Merino J., Ma W., Lo C.-H., Polidori L. (2021). Racial and ethnic differences in COVID-19 vaccine hesitancy and uptake. MedRxiv.

[47] Wagner A.L., Masters N.B., Domek G.J., Mathew J.L., Sun X., Asturias E.J., Boulton M.L. (2019). Comparisons of vaccine hesitancy across five low-and middle-income countries. Vaccines.

[48] Aw J., Seng J.J.B., Seah S.S.Y., Low L.L. (2021). COVID-19 vaccine hesitancy—A scoping review of literature in high-income countries. Vaccines.

[49] Household Pulse Survey Data Tables. https://www.census.gov/programs-surveys/household-pulse-survey/data.html.

[50] Lindemer E., Choudhary M., Donadio G., Pawlowski C., Soundararajan V. (2021). Counties with lower insurance coverage are associated with both slower vaccine rollout and higher COVID-19 incidence across the United States. MedRxiv.

[51] Juhn Y.J., Sauver J.S., Katusic S., Vargas D., Weaver A., Yunginger J. (2005). The influence of neighborhood environment on the incidence of childhood asthma: A multilevel approach. Soc. Sci. Med..

[52] Bureau U.C. (2006). American Housing Survey for the United States: 2005 Current Housing Reports.

[53] Jacobs D.E. (2011). Environmental health disparities in housing. Am. J. Public Health.

[54] Holden C.D., Chen J., Dagher R.K. (2015). Preventive care utilization among the uninsured by race/ethnicity and income. Am. J. Prev. Med..

